# Platelets in Neurodegenerative Conditions—Friend or Foe?

**DOI:** 10.3389/fimmu.2020.00747

**Published:** 2020-05-05

**Authors:** Odette Leiter, Tara L. Walker

**Affiliations:** Queensland Brain Institute, The University of Queensland, Brisbane, QLD, Australia

**Keywords:** platelets, neurodegeneration, neuroinflammation, brain function, neuroimmune crosstalk

## Abstract

It is now apparent that platelet function is more diverse than originally thought, shifting the view of platelets from blood cells involved in hemostasis and wound healing to major contributors to numerous regulatory processes across different tissues. Given their intriguing ability to store, produce and release distinct subsets of bioactive molecules, including intercellular signaling molecules and neurotransmitters, platelets may play an important role in orchestrating healthy brain function. Conversely, a number of neurodegenerative conditions have recently been associated with platelet dysfunction, further highlighting the tissue-independent role of these cells. In this review we summarize the requirements for platelet-neural cell communication with a focus on neurodegenerative diseases, and discuss the therapeutic potential of healthy platelets and the proteins which they release to counteract these conditions.

## Introduction

**Figure 1:**
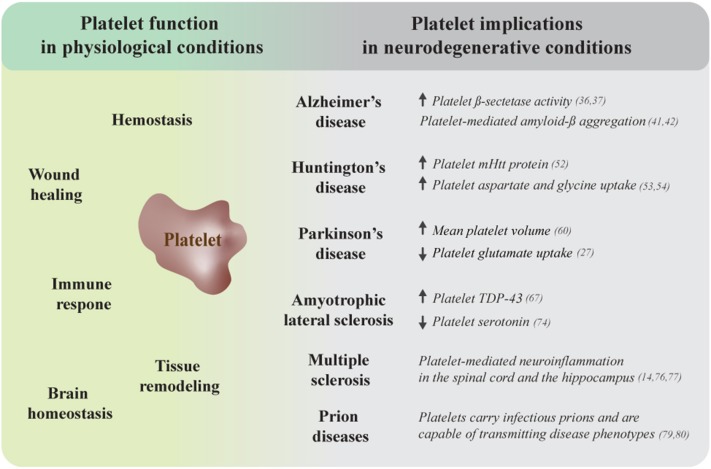
Platelet dysfunction is associated with several neurodegenerative disorders. Platelets are complex cells that exert numerous regulatory functions under physiological conditions, ranging from their traditional roles in hemostasis and wound healing to fundamental contributions to immune and tissue remodeling processes and brain homeostasis (left side). Platelet dysfunction, including mitochondrial abnormalities, is a common observation during neurodegeneration. The right side of this figure summarizes additional platelet-related impairments that link these cells to several neurodegenerative conditions. mHtt, mutant huntingtin protein; TDP-43, TAR DNA-binding protein of 43 kDa.

Platelets are small anucleate blood cells that have been gaining recognition as important mediators of several regulatory processes. Emerging research has identified novel functions that reach well beyond the traditional role of platelets in hemostasis and wound closure, revealing them to be crucial players during immune responses and tissue remodeling processes. We have recently summarized the evidence highlighting the capacity of platelets to contribute to brain homeostasis under physiological circumstances (1). Whereas, their versatile functions make platelets important regulators of cellular processes under normal conditions, platelet dysfunction is linked to a number of pathologies, including neurodegeneration. In the following review we briefly discuss the prerequisites of intercellular communication between platelets and cells from the central nervous system and summarize the research that demonstrates the involvement of impaired platelet function in several neurodegenerative conditions, including Alzheimer's disease (AD), Huntington's disease (HD), Parkinson's disease (PD), amyotrophic lateral sclerosis (ALS), multiple sclerosis (MS), and prion diseases (Figure 1). Finally, we highlight the emerging role of platelet preparations in the development of therapeutic interventions for the treatment of neuropathologies.

## Platelets—The Diverse Properties of a Small Blood Cell

Until recently, platelets were primarily known for initiating coagulation following tissue injury and endothelial disruption. Although the platelet count in healthy humans ranges from 150,000 to 400,000 platelets per microliter of blood (2), only a small fraction of these (about 10,000 platelets per microliter) are necessary to act during hemostasis (3), supporting reports that platelets also exert other functions. Platelets are produced in the bone marrow by megakaryocytes which equip them with cytoplasm, including messenger ribonucleic acid (mRNA), mitochondria and secretory vesicles such as lysosomes, dense granules and α-granules, before they are released into the blood. Mouse and human platelets are functionally similar (4) and have short lifespans of 4–5 days and 8–12 days, respectively (5). However, a recent study found that platelets can return to the circulation following activation by thrombotic and immunological stimuli, suggesting that their lifespan could be longer than traditionally thought and that their elimination is not a direct consequence of the activation process (6). Platelet activation is required to fulfill particular functions; however, the outcome is specific to the trigger which initiates the activation. The most common platelet responses to activating stimuli include changes in shape, the upregulation of cell surface molecules, protein synthesis from mRNA, endo- and exocytosis, and the release of molecules from granule contents. In particular, the context-dependent secretion from α-granules, which provide a storage compartment for abundant bioactive molecules including growth and coagulation factors, chemokines, immune molecules and adhesion molecules, is highly regulated. Consequently, the stimulation of platelet preparations with three common agonists, adenosine diphosphate, collagen and thrombin receptor-activating peptide, results in distinct protein secretion profiles (7). In another study, it was shown that subpopulations of α-granules exist, in which proteins are stored in distinct clusters such as pro- or anti-angiogenic protein clusters (8). The selective release of these granule subtypes was triggered by the stimulation of different receptors with specific agonists, indicating that α-granule cargo is secreted in a context-dependent manner to either inhibit or promote angiogenesis as required (8). The finely tuned mechanisms whereby bioactive molecules are released from platelets represent a crucial asset in orchestrating regulatory processes across different tissues. However, disturbances in the regulation of platelet responses or hyperactivation of platelets have implications in numerous diseases, including during neurodegenerative conditions, as described in more detail below.

## Platelets are Experts in Cell-Cell Communication

Platelets can communicate with other cell types in multiple ways, with their flexibility and mechanistic diversity suggesting that they likely act as inter-tissue messengers, including between blood and brain cells. Although the secretion of bioactive molecules from α- and dense granules represents a likely route of intercellular communication, additional mechanisms via which platelets may support crosstalk between the brain and the systemic environment are possible. Platelets release extracellular vesicles containing active cytoplasm components such as exosomes and microparticles (9). Both represent common ways of intercellular communication between organs and tissues in health and disease. Platelet exosomes and microparticles can also contain microRNAs, which when dysregulated are involved in various neurodegenerative disorders, including AD, PD, MS, HD, and ALS (10). Moreover, platelet-released particles, as well as platelets themselves which measure ~0.5 μm in diameter in mice (5) and from 1 to 5 μm in humans (11), are small enough to travel deep within the microcapillaries that span the brain. Thus, platelets and their released factors could interact with specific receptors in the cerebral vasculature to exert local, receptor-mediated effects. In conditions where the vascular integrity is altered or disturbed direct interactions with neural cells are possible. Platelet activity has been observed within the brain parenchyma following lesion (12) and stroke (13), as well as in the brain of experimental autoimmune encephalomyelitis (EAE)-induced mice (14). Furthermore, a direct interaction between platelets and neuronal cells has been reported, as they can bind central nervous system-specific glycolipid structures that are present in the lipid rafts of neuronal processes (15). This interaction was recently shown to stimulate the growth of new dendritic spines (16). The proposed mechanisms via which platelets communicate with neural cells have been discussed in more detail elsewhere (1); however, these mechanisms could also influence neural cell properties under neurodegenerative conditions. Moreover, as reviewed below, platelets exhibit neuron-like properties that further facilitate crosstalk between these cells and the central nervous system.

## The Neuron-like Properties of Platelets—Bridging the Gap Between the Systemic Environment and Brain Pathologies?

Despite their distinct location and function, platelets and neural cells are remarkably similar, suggesting a potential path of cross-communication between the systemic environment and the brain. In particular the intercellular storage compartments in neurons, which contain neuropeptides, neurohormones and neurotransmitters, are comparable to platelet granules, including the use of similar vesicle trafficking mechanisms. Platelet dense granules resemble the small dense-core synaptic vesicles of neurons in terms of their serotonin and adenosine triphosphate contents, among other features, whereas the large dense-core vesicles of neurons are comparable to platelet α-granules. Both storage compartments carry a large variety of bioactive peptides, and stimulus-specific secretion processes are observed in both neurons (17) and platelets (8). This indicates that the strict regulation of selective exocytosis is a conserved mechanism in both cell types (18). Platelet and neuronal exocytosis are both triggered by an increase in the internal calcium concentration (19), leading to the rapid activation of the secretory machinery. Moreover, the mechanism whereby the internal vesicles fuse with the plasma membrane is highly conserved, occurring via specific docking molecules such as SNAREs, VAMPs and syntaxins (19). Other review papers have discussed the molecular similarities between platelets and neuronal cells in more detail (18–20), and have proposed that platelets could even be considered “neuronal cells” themselves, with the interaction between platelets and T cells representing a novel “neuroimmunological” synapse in the periphery (20). Likewise, platelets could act as messengers, transferring signals between the peripheral environment and brain cells. We have shown that platelet-rich plasma has direct stimulating effects on a pure population of flow cytometry-isolated hippocampal dentate gyrus-derived neural precursor cells *in vitro*, and that mice which have been depleted of platelets fail to show the expected exercise-induced increase in neural precursor cell proliferation *in vivo* (21). This work suggests that platelet-neural stem cell communication is an important regulatory mechanism in these brain cells, although the precise molecular mechanisms underlying this communication are still unclear.

Platelets carry several neurotransmitters that are essential for the intercellular communication between brain cells, including γ-aminobutyric acid (GABA), glutamate, serotonin, epinephrine, dopamine, and histamine. This suggests that platelets can send and receive signals to and from the nervous system and may act as an important relay between the brain and peripheral organs. The monoamine neurotransmitter serotonin is stored in dense granules, and peripheral serotonin release-associated regulatory functions of platelets have been described (6, 22). Although the peripheral and central nervous system serotonergic systems are thought to be separated, platelets release serotonin in response to brain-specific glycolipid structures, which are integrated into the lipid rafts of neurons and astrocytes (15). Such interactions could occur in conditions in which cerebral microvessels become leaky, including during neurodegenerative diseases (23), suggesting that platelets could act as communicators between blood and brain. This hypothesis becomes more cogent when considering the two major neurotransmitters GABA and glutamate, both of which are taken up by platelets (24). Glutamate is the most abundant excitatory neurotransmitter in the brain, and substrate-induced glutamate uptake has been demonstrated in human platelets, likely via specific glutamate receptors (25), similar to what is observed in neuronal cells (26). Platelets express various glutamate receptor subtypes and exhibit high affinity glutamate uptake activity, a process which is impaired in disorders such as PD (27), AD (28) and ALS (29). GABA, an inhibitory neurotransmitter, is crucial for healthy brain function, with perturbances in GABA receptor signaling being associated with neurodegenerative conditions [reviewed in Kim et al. (30)]. Platelets carry considerable amounts of GABA, although the concentration is 30% lower than that found in neurons (31). In both neurons and platelets GABA is metabolized by GABA transaminase (31). Moreover, similar to neurons, platelets appear to take up GABA in a substrate-induced manner, with an *in vitro* study reporting that the GABA concentration in platelets is negligible when the peripheral benzodiazepine receptor blocker PK11195 is present in the cell culture medium (31).

Given these similar mechanisms of neurotransmitter uptake and metabolism, platelets have been suggested as a model system of glutamate and GABA transport in patients suffering from neurodegenerative conditions (25, 31). A more recent review article has extended these concepts to other conserved mechanisms between platelets and neurons that are associated with neurodegenerative diseases, with platelet dysfunction mirroring the abnormalities observed in neurons (32). However, to date it is unclear whether platelet dysfunction occurs first or whether functional impairments in platelets arise as a consequence of other defects that occur during neurodegenerative processes.

## Platelets in Neurodegenerative Conditions

It is becoming clear that neurodegenerative diseases do not solely involve cells and tissue of the central nervous system, but rather that systemic influences also play a fundamental role in the development and exacerbation of brain pathologies. As discussed above, platelets are of particular interest as important mediators of this two-way relationship. Several review papers have concluded that these blood cells can serve as potent systemic biomarkers of neurodegenerative diseases, mirroring the pathological phenotypes of neural cells (32–34). In this section we describe the studies that link platelets to neurodegenerative conditions, with a particular focus on platelet dysfunction in these disorders (summarized in Table 1).

**Table 1:** Platelet abnormalities linked to neurodegenerative conditions.

**Condition**	**Implication of platelets**	**Species/model**	**Reference**
Alzheimer's disease	Increased platelet activation	Human	(35)
	Increased platelet β-secretase activity	Human	(36, 37)
	Platelet hyperactivity	APP23 mice	(38)
	Increased adhesion to subendothelial matrix components	3xTg-AD mice	(39)
	Platelet inclusions in cerebral blood vessels	APP_SweDI mice	(40)
	Platelets enhance formation of amyloid-β aggregates in cerebral vessels	APP23 mice	(41)
	Platelets promote neuroinflammation and vessel damage	APP_SweDI mice	(42)
	APP and amyloid-β influence platelet function	Human/APP-KO, C57BL/6 and APP23 mice	(41, 43–51)
Huntington's disease	Increased platelet mHtt protein levels	Human	(52)
	Increased platelet aspartate and glycine levels	Human	(53, 54)
	Platelets promote blood brain barrier permeability	Human	(52)
	Impaired platelet adenosine A receptor signaling	Human	(55, 56)
	Impaired platelet nitric oxide metabolism	Human	(57)
	Elevated platelet mitochondrial monoamine oxidase activity	Human	(58, 59)
Parkinson's disease	Increased mean platelet volume	Human	(60)
	Decreased platelet glutamate uptake	Human	(27)
	Reduction in vesicular monoamine transporter 2 mRNA	Human	(61)
	Platelet mitochondrial dysfunction	Human/ Cybrid model	(62–66)
Amyotrophic lateral sclerosis	Increased platelet TDP-43 levels	Human	(67)
	Reduced complex IV activity in platelet mitochondria	Human	(68)
	Altered platelet mitochondrial membrane potential	Human	(69)
	Altered platelet mitochondrial morphology	Human	(70)
	Altered platelet activation and morphology	Human	(70)
	Enlarged mitochondria, degenerating mitochondrial vacuoles and neurofilament aggregations	Cybrid model	(71–73)
	Decreased platelet serotonin levels	Human	(74)
Multiple sclerosis	Increased platelet activation	Human	(75)
	Platelets drive neuroinflammation in the spinal cord	EAE mice	(76, 77)
	Platelet-neuron associations are associated with neuroinflammation in the hippocampus	EAE mice	(14)
	Altered serotonin release from dense granules	Human/EAE mice	(78)
Prion diseases	Platelets carry infectious prions	Deer	(79)
	Platelets are capable of transmitting disease phenotypes	Deer and sheep	(79, 80)

*AD, Alzheimer's disease; APP, amyloid precursor protein; EAE, experimental autoimmune encephalomyelitis, mHtt, mutant huntingtin protein; TDP-43, TAR DNA-binding protein of 43 kDa*.

### Alzheimer's Disease

AD is a slowly developing progressive form of dementia that is accompanied by unpredictable behavior, lack of enthusiasm and memory loss. The neuropathological hallmarks of AD include neuronal and synaptic loss, neuroinflammation, the formation of intracellular neurofibrillary tangles and the deposition of amyloid-ß in brain tissue and cerebral vessels. Increasing evidence has linked platelet dysfunction to this disease, in particular in the context of amyloid-ß secretion from platelets.

Although neural cells, including astrocytes and neurons, produce and secrete amyloid-ß (81), the peptide can also be released by activated platelets (82). Platelets have been suggested to be the primary source of amyloid-ß peptide in the blood (83). The cells produce this peptide through the cleavage of its precursor protein, amyloid precursor protein (APP), which is abundantly present in platelets and is secreted following platelet activation, similar to its metabolite amyloid-ß (82, 84, 85). Both APP and amyloid-ß peptide are associated with platelet functions. Whereas, APP is involved in the regulation of thrombosis and coagulation (46–48), amyloid-ß peptide has the ability to promote platelet activation (41, 49–51), adhesion (43, 48, 50), aggregation (47, 48) and to induce reactive oxygen species generation (45, 51).

Rather than alterations in platelet count or size, changes in platelet activation appear to play a prominent role in AD, with increases in activation detected in the blood of AD patients, likely as a result of increased lipid peroxidation (35). Similarly, platelets have been shown to be hyperactive in aged APP23 transgenic mice, a model of AD (38). A subsequent study confirmed abnormalities in platelet function in a more complex mouse model of AD, 3xTg-AD mice, with increased platelet adhesion to components of the subendothelial matrix and accelerated thrombus formation, although the platelet count remained unchanged (39). In patients with mild cognitive impairment and AD, the activity of ß-secretase, one of the major enzymes required for the cleavage of APP, is significantly increased in the membranes of platelets (36, 37), suggesting further platelet-related systemic changes during the disease.

A recent parabiosis study, in which the blood circulation of APPswe/PS1dE9 transgenic AD model mice was connected with that of their wildtype counterparts demonstrated that human amyloid-ß originating from the transgenic mice accumulated in the brains of their healthy littermates, forming amyloid-ß plaques and amyloid angiopathy following 12 months of parabiosis (86). Moreover, the parabiotic wildtype mice exhibited impaired long-term potentiation in the hippocampal cornu ammonis 1 area, suggesting a reduction in synaptic plasticity, which is thought to underlie deficits in learning and memory (86). Although this study did not investigate the origin of the blood-derived amyloid-ß, the authors suggested platelets as a likely source.

Prior to amyloid-ß plaque formation, platelet inclusions in cerebral blood vessels are among the first symptoms to appear in the brains of APP_SweDI AD model mice (40). Another study demonstrated that platelets enhance the formation of amyloid-ß aggregates in the brain vasculature and that amyloid-ß itself can activate platelets (41). In the same study, the plaque burden of cerebral vessels in APP23 mice was significantly reduced following a 3-month treatment with clopidogrel, a known inhibitor of platelet activation (41). Interestingly, a trend toward reduced plaque formation was also observed within the hippocampus, a brain region which is crucial for learning and memory and is profoundly affected by AD (41). More recent work has shown that platelets isolated from APP_SweDI mice promote vessel damage and neuroinflammation in the healthy mouse brain, leading to amyloid-ß-like immunoreactivity at the damaged vessel sites (42). Together these data suggest that hyperactive AD platelets release and interact with amyloid-ß specifically at sites of vessel damage, thereby accelerating the progression of the disease (38, 39, 41, 42). This is in line with work suggesting that AD may, at least in part, be a slowly developing thrombohemorrhagic disorder (87, 88), highlighting the need to expand research beyond the brain and consider treatment of the systemic environment in AD patients. In this regard, platelets represent a potential target, with a reduction in platelet count being suggested as a means to counteract the overproduction of amyloid-β (87).

An interesting alternative theory is that amyloid-ß release represents a defense mechanism against septic agents (89, 90). Recent research indicates that amyloid-ß may be a normal component of the innate immune system, protecting individuals against microbial and viral infection (91–94). Given the emerging evidence that platelets act as fundamental immune cells, including in the brain [summarized in Leiter and Walker (1)], they could accumulate at damaged cerebral vessel sites and release amyloid-ß as a defense peptide. This is in line with a study which suggests that the release of amyloid-ß from platelets is triggered by pre-existing tissue damage and inflammation and represents a natural protective mechanism against infection during thrombosis (92). However, the platelet hyperactivity that is associated with AD may lead to the overproduction of amyloid-ß, thereby exacerbating inflammation and eventually promoting the development of plaque formation.

Although the studies described above focused on amyloid-ß, this peptide does not represent the only known link between platelets and AD, with other investigators examining the involvement of neurofibrillary tangles and impaired neurotransmitter homeostasis. These studies have been reviewed elsewhere (95).

### Huntington's Disease

HD is a hereditary autosomal dominant neurodegenerative disorder caused by a CAG repeat expansion in exon 1 of the huntingtin gene, resulting in the production of a mutant huntingtin protein (mHtt). This protein accumulates in neurons, thereby leading to their eventual death and a progressive loss of motor and cognitive functions. Extensive research has shown that a number of cell subpopulations in the blood are altered in HD patients, with platelets having the highest levels of mHtt (52).

The platelets of HD patients exhibit a number of abnormalities, including aberrant amplification of adenosine A receptor (A_2A_R) signaling (55, 56). Given that the A_2A_R is expressed in GABA/enkephalin spiny neurons, it has been proposed that it may play a role in HD pathogenesis. Other studies have also reported a correlation between the density of A_2A_R in platelets and the rate of disease, age at onset and CAG repeat expansion (55, 96). However, whether or not A_2A_R activity provides a useful biomarker remains to be determined.

Dysfunction of the nitric oxide /nitric oxide synthase pathway and monoamine oxidase (MAO) have also been suggested to be critical contributors to HD pathology. Nitric oxide metabolism has been found to be dysregulated in platelets during the late stages of HD progression (57), and MAO activity has been associated with neuronal damage in a number of degenerative conditions. MAO is a mitochondrial enzyme that catalyzes the oxidative deamination of monoamines such as dopamine. MAO exists in the MAO-A and MAO-B isoforms. Whereas, some cell types express both isoforms, only MAO-B is found in platelets. Significantly elevated platelet MAO activity has been observed in HD patients during disease progression (58, 59), with the levels negatively correlating with the clinical response to drug treatment (97).

A proposed model of HD pathogenesis is the “excitatory hypothesis,” based on the observation that excitatory amino acids and N-methyl-D-aspartate receptor agonists, including aspartate and glutamate, recapitulate the striatal neuron degeneration observed in HD (98). Although early studies found no differences in glutamate and aspartate activity between normal and HD platelets (99, 100), later studies have reported significantly increased aspartate and glycine in HD platelets (53, 54).

Mitochondrial dysfunction has also been implicated in the pathogenesis of HD. A significant decrease in mitochondrial complex I activity per platelet was observed when patients were grouped according to disease severity; however, when normalized to mitochondrial DNA content, no differences were detected (101). In contrast, an earlier study found no difference in platelet mitochondrial complex activity in HD patients (102). Given the relatively small group sizes, further data are required to determine whether mitochondrial function in platelets provides a useful biomarker of HD. However, increased mitochondrial-dependent apoptosis has also been reported in HD cybrids (103).

Platelets are also important in maintaining normal vascular integrity (104). Recently, an initial study investigating the potential impact of mHtt on platelet function showed that platelets can promote blood brain barrier permeability in HD, pointing toward their potential contribution to disease pathogenesis (52).

### Parkinson's Disease

PD is a degenerative disorder caused by the loss of dopaminergic neurons in the substantia nigra, thereby resulting in an impairment in motor and cognitive functions. Although the cause of sporadic PD, the most common form of the disease, is unknown, one major causal factor is mitochondrial dysfunction. This was first suggested by the finding that 1-methyl-4-phenyl-1,2,3,6-tetrahydropyridine (MPTP), a neurotoxin that selectively kills dopaminergic neurons, acts by inhibiting complex I of the electron transport chain (105). A plethora of studies have reported reduced complex I activity in the platelets of patients with PD (62–64), although it should be noted that other studies did not find such alterations (65, 66). Supporting the former observation, a PD cybrid model in which mitochondrial DNA from PD platelets was expressed in rho 0 human teratocarcinoma cells showed a reduction in complex I activity (106, 107). In addition, 1-methyl-4-phenyl-pyridinium ion MPP(+), the metabolite of MPTP, was shown to induce adenosine triphosphate depletion in platelets and attenuate platelet aggregation and activity, providing a potential mechanism underlying the anti-aggregation effect observed in PD patients (108).

Several studies have suggested that MAO also plays an important role in MPTP toxicity and the etiology of PD. Increased MAO-B activity has been observed in PD patients (109–111), potentially due to a G/A single nucleotide polymorphism in intron 13 which results in a splicing enhancer that stimulates intron 13 removal efficiency (110). However, the data concerning platelet MAO-B activity in PD patients are not consistent, with other studies reporting that platelet MAO-B activity is unchanged in PD patients (112, 113).

A number of other alterations in the platelets of PD patients have also been suggested as potentially useful biomarkers. These include a reduction in vesicular monoamine transporter 2 mRNA (61), an increase in mean platelet volume (60), and decreased glutamate uptake (27).

### Amyotrophic Lateral Sclerosis

ALS is a fatal neurodegenerative disorder that is characterized by progressive and selective loss of motor neurons in the brain and spinal cord. Patients suffer from progressive muscle weakness and paralysis of their voluntary muscles, ultimately leading to respiratory failure and death. There is accumulating evidence that in addition to affecting motor neurons, ALS also affects platelets.

Almost all ALS cases (~97%) are characterized by pathology due to the TAR DNA-binding protein of 43 kDa (TDP-43) (114, 115). In diseased neurons, TDP-43 is relocated from its normal nuclear location to the cytoplasm, where it is phosphorylated and ubiquitinated, subsequently aggregating to form insoluble intracellular inclusions (115). A recent study found that the TDP-43 levels in platelets from patients with sporadic ALS are significantly higher than those of non-ALS age-matched controls (67). Interestingly, the TDP-43 levels in platelets tended to increase with disease progression, although a larger cohort of patients is required to confirm this observation (67).

Mitochondrial abnormalities, particularly impairments of complex IV (cytochrome c-oxidase) activity, have been implicated in ALS, although the exact role of mitochondrial dysfunction remains unclear. In addition to mitochondrial dysfunction in motor neurons of ALS patients, mitochondrial changes have also been reported in muscle, liver and blood cells, suggesting systemic involvement (116–118). Complex IV activity was found to be decreased in platelets from ALS patients in a small case-control study (68). Interestingly, the cellular mitochondrial content increased, indicating a potential compensatory mechanism (68). Further supporting the notion of mitochondrial dysfunction, a change in the mitochondrial membrane potential has been reported in platelets from ALS patients (69), as well as changes in the ultrastructure and morphology of platelets and their mitochondria (70). This is in line with an earlier study which observed platelet activation and morphological changes in ALS platelets (119). ALS cybrids (platelets fused to the rho neuronal cell lineage) also show similar cytoskeletal deformities to those found in ALS patients and transgenic superoxide dismutase 1 mice, including enlarged mitochondria, degenerating mitochondrial vacuoles and neurofilament aggregations (71–73). Despite these links between platelet mitochondrial dysfunction and ALS, larger cohort studies are required to conclusively determine whether mitochondrial function can be used as a biomarker for ALS.

Thrombospondin is a glycoprotein that is released from platelet α-granules following thrombin-induced platelet activation. Changes in blood thrombospondin levels have been detected in a number of pathological conditions, including a marked increase in thrombospondin deposition in the muscles of ALS patients (120, 121). The neurotransmitter serotonin is also decreased in the brain and spinal cord of ALS patients (122, 123). Platelets are a major source of serotonin and platelet serotonin levels have been shown to be significantly lower in ALS patients and to positively correlate with patient survival (74). However, the cause of this decrease in serotonin remains elusive. Glutamate excitotoxicity has also been implicated in the pathogenesis of the disease. Platelets contain a glutamate uptake system and express components of the glutamate-glutamine cycle, including the excitatory amino acid transporter 2 and glutamine synthetase. Increased glutamine synthetase, but normal excitatory amino acid transporter 2 expression, has been reported in the platelets of ALS patients (124). However, given that this finding is in contrast to an earlier study which reported a reduction in glutamate uptake in ALS patients (29), these data need to be confirmed.

### Multiple Sclerosis

MS is an inflammatory disease, where the immune system attacks the myelin sheaths that cover nerve axons in the spinal cord and brain. The resulting nerve damage leads to communication deficits between the brain and other tissues, and depending on the affected nerves provokes a range of symptoms, including impairments in vision, deficits in motor control of the arms and legs and neuropsychological symptoms such as depression and memory loss. To date, there is no known cure for MS, as the underlying cause is still unknown.

A few studies targeting platelets and their involvement in MS and its mouse model, EAE, have shown that these conditions are associated with abnormalities in platelet function. One of these investigations found increased platelet activation in the blood of clinically stable relapsing-remitting MS patients who had not yet received treatment (116). This was evidenced by significantly larger numbers of CD62P-positive platelets and CD41-positive platelet microparticles (75). Subsequent evidence in EAE mice revealed that platelets exacerbate the development of the disease via the recruitment of leukocytes to the neural tissue (76). A more recent study cemented the involvement of platelets in EAE, demonstrating that platelets not only aggravate (76) but also drive neuroinflammation in the spinal cord (77). Possible mechanisms via which platelets could exacerbate the pathophysiology of MS are discussed in a review by Wachowicz et al. with one interesting concept being an impaired antioxidant mechanism in combination with inflammation-induced platelet activation as an additional source of reactive oxygen species to further accelerate tissue damage (125). Moreover, the secretion of serotonin from dense granules has been shown to modulate immune cell responses in a stage-depended manner. During the early stages of EAE and MS, high levels of platelet-released serotonin stimulate the proliferation and differentiation of pathogenic T cell subsets, thereby promoting proinflammatory responses (78). During later phases of the disease, however, platelets exhibit reduced serotonin levels and appear to suppress T cell activation and central nervous system inflammation (78).

Recent work investigating the brains of EAE-induced mice demonstrated that platelets were also present in the parenchyma of the hippocampus, including in the fimbria and in close proximity to neuronal cell bodies in the dentate gyrus and CA1 region (14). This phenotype was associated with the formation of a neuroinflammatory environment, supposedly due to platelet-neuron associations (14). However, this occurred in the absence of inflammatory cell infiltration, further highlighting the role of platelets in the initiation of EAE (14). In the same study, the pro-inflammatory environment in the hippocampus of EAE-induced mice, as well as their increased anxiety-like behavior, were improved following platelet depletion with polyclonal anti-platelet glycoprotein Ib α chain antibodies, suggesting that platelets could serve as a potential target for the amelioration of the symptoms of MS (14).

### Prion Diseases

Prion proteins (PrPs) comprise a class of amyloid-forming proteins, with some isoforms being associated with a group of fatal neurodegenerative diseases termed transmissible spongiform encephalopathies. Once diagnosed, these conditions progress rapidly and are characterized by the chronic deterioration of physical and mental abilities, including profound memory impairments. The scrapie isoform of PrP is an abnormal, misfolded, protease-resistant isoform (126, 127) which is believed to be responsible for transmissible spongiform encephalopathies. Although considered transmissible, the paths through which prion diseases spread are unknown, with the transfusion of blood from infected donors presenting a concern.

Cellular PrP (PrP^c^) is carried by blood cells, including platelets, in which PrP^c^ is present on the membranes of α-granules (128, 129). Following activation, PrP^c^ can be released from activated platelets, mainly in the form of microparticles and exosomes (128). The function of PrP^c^ under these circumstances is unknown, although it has been reported that the protein is unlikely to play a role in the aggregation or adhesive actions of activated platelets (128). The release of microparticles and exosomes represents a major route of intercellular communication, including crosstalk between platelets and neural cells (1). This suggests that in the course of transmissible spongiform encephalopathies, the less soluble scrapie prion isoform could be carried and released from activated platelets thereby contributing to the infection of the brain and the transmission of the disease through blood transfusion (128). Other work has confirmed that platelets and B cells in the blood of deer, infected with chronic wasting disease carry infectious prions, and are substantially involved in transmitting the disease phenotype (79). In a sheep model of variant Creutzfeldt Jakob disease, the disease could be transmitted through several blood components, such as whole blood, plasma, red blood cells, buffy coat and platelets (80). These data from animal studies suggest a high probability that spongiform encephalopathies are transmissible through blood (79, 80), even in pre-clinical stages of the disease (80). However, only a few cases suggest this possibility in humans, where the lack of a causal link between blood transfusions and the development of prion diseases makes it difficult to draw a conclusion (130–132).

## Platelets—A Novel Therapeutic Avenue for the Treatment of Neurodegenerative Conditions?

Impairments in platelet function are a common observation in neurodegenerative disorders; however, healthy platelets and their secreted factors also represent a possible approach for the development of therapeutic interventions for the treatment of neurodegenerative conditions. Among the primary applications are the use of platelet lysate and platelet-rich plasma, both of which are easy to obtain from immune-compatible healthy donors. The beneficial effects of platelet-rich plasma treatment are likely to be attributable to the abundant variety of growth factors that platelets carry in their granules. Neural and glial cells express surface receptors for a range of these growth factors, including vascular endothelial growth factor, epidermal growth factor, fibroblast growth factor-2, platelet-derived growth factor, brain-derived neurotrophic factor, platelet factor 4, transforming growth factor-ß, insulin-like growth factor-1, connective tissue growth factor and bone morphogenetic protein-2,−4, and−6, suggesting a fundamental role of platelets in tissue growth and regeneration, including in the brain (133–135). Moreover, human platelet lysate comprises a plethora of growth factors, including those with neuroprotective properties. Although emerging research has shown promising results, diverse protocols for the isolation of platelet-rich plasma and platelet lysates exist, resulting in products which contain variable ranges of growth factors (136). Moreover, novel protocols are continuously being published, describing optimized preparations for specific use in different applications (137–139). These factors therefore represent an important consideration when evaluating study outcomes and planning future clinical trials across different fields.

### Platelet-Rich Plasma

Platelet-rich plasma can easily be prepared from whole blood using a slow centrifugation speed and physiological washing buffers that support platelet purification. This method achieves a nearly pure population of platelets [>99.99% purity (140)], and the platelet preparation can be used immediately or stored. However, upon freezer storage and subsequent thawing of the samples, a substantial number of cells will be lyzed, leading to the release of growth factors from platelet granules. These are also present in frozen/thawed platelet-rich plasma preparations, making them a physiological cocktail of intact cells and released bioactive molecules.

Beneficial therapeutic effects of platelet-rich plasma treatment have been reported in numerous tissues, including during burn healing (141, 142), cartilage repair (143) and healing following dermal injuries (144). Other studies have demonstrated that platelet-rich plasma treatment enhances the recovery of peripheral nerves following injury, including cavernous nerve injuries (145) and damage of the facial (146) and sciatic (147) nerves. Moreover, platelet-rich plasma injections into the injured spinal cord of rats have been shown to promote locomotor recovery, local angiogenesis and neuronal regeneration (148). Another study in mice suggested the therapeutic use of platelet-rich plasma in neuroinflammatory central nervous system diseases, as platelet-rich plasma treatment considerably improved the clinical symptoms in the EAE mouse model of MS (149). This effect was accompanied by significantly lower gene expression and a decrease in the protein levels of inflammatory markers in the lumbar parts of the spinal cord, including the microglial marker Iba1 and the pro-inflammatory cytokine interleukin 1-β, as well as the reduced infiltration of inflammatory cells (149). The platelet-rich plasma injection also protected the cells from demyelination in the affected area (149). Other studies which used the plasma rich in growth factors Endoret^®^ technology to isolate platelet-rich plasma from human blood have demonstrated that treatment with these preparations significantly reduces amyloid-β plaque density in the hippocampus and improves cognitive function in APP/PS1 AD model mice (150). Another study complemented this finding showing that the same preparations enhanced adult neurogenesis in the hippocampus of APP/PS1 mice, a process known to be affected during AD, and that this enhancement was likely due to a reduction in amyloid-β-mediated neurotoxicity (151). The same method also promoted neuronal survival and diminished the inflammatory responses in a mouse model of PD, as well as reducing the associated motor impairments (152). These data suggest that platelet-rich plasma treatment represents a promising approach which could be applied to several neurodegenerative disorders.

### Platelet Lysate

Similar to platelet-rich plasma, platelet lysate can be easily obtained from whole blood samples. Platelets are first enriched by centrifugation steps, followed by freezing and thawing of the samples. An additional centrifugation step then separates the freeze/thaw-triggered secreted platelet factors, which constitutes the platelet lysate, from the remaining cell debris.

Given their essential role in wound healing and tissue repair, platelet lysates are being investigated as a therapy for a number of neurodegenerative diseases. Human platelet lysates have been investigated as a novel biotherapy for ALS and PD patients. In an NSC-34 cell-based model of ALS, human platelet lysates conferred a neuroprotective effect against staurosporine-induced apoptosis and menadione-induced oxidative stress, indicating that neuronal loss can be diminished by platelet factors in those conditions (153). In a Lund human mesencephalic cell-based model of PD, pre-treatment of the cells with human platelet lysates also protected again erastin-induced ferroptotic cell death (153). The authors further optimized the isolation protocol to produce platelet lysate preparations which are more enriched for neurotrophins and at the same time depleted of plasma proteins, thereby preventing potential adverse thrombotic effects during *in vivo* applications (137). Following intranasal administration of the optimized platelet lysate, obvious protective effects were observed on dopaminergic neurons in the substantia nigra and the striatum of PD model mice (137). The intranasally administered platelet factors were also found in several other regions of the brain, including the striatum, olfactory bulb, and cortex (137), making this treatment method a promising tool for application in various neurodegenerative conditions.

Although we have not addressed stroke and other brain injuries in this review, human platelet lysate treatment has also been shown to produce positive outcomes in these conditions. Following stroke, human platelet lysate injections into the lateral ventricles of rats had neuroprotective effects (154). The platelet lysate-treated rats exhibited a larger number of proliferating neural precursor cells in the subventricular zone, accompanied by increased angiogenesis (151). They also displayed lower motor function deficits (154). Another study demonstrated that administrating human platelet lysate decreased apoptosis and stimulated the survival of proliferating neural precursor cells in the same brain region after a lysolecithin-induced demyelination lesion in the corpus callosum (12), further suggesting a neuroprotective role of platelets after cerebral damage.

### Platelets and Platelet Microparticles—Potential Vehicles for the Delivery of Therapeutic Drugs?

In addition to platelet-rich plasma and platelet lysate preparations, an interesting approach is emerging, whereby platelets are used as a physiological vehicle to deliver molecules to target regions that might otherwise be difficult to access. With their context-dependent and specific cell-cell communication capacity, platelets could serve as a selective and non-toxic drug delivery system in order to target specific cells and tissues. This approach has been extensively discussed previously, with a particular focus on the use of platelets to deliver chemotherapeutic agents to tumors (155). However, this novel strategy still requires additional studies to confirm its efficacy. Microparticles, which are released by platelets upon activation, have also been proposed as a natural delivery system for drugs (155, 156). The majority of all microvesicles in the blood are platelet-derived (157), indicating a vital contribution of platelets to intercellular communication. Platelet microparticles, which are 0.1–1 μm in diameter, are shed from the plasma membrane (158) and contain cytoplasm, microRNA, mRNA, lipids and proteins. These can be transferred to other cells, thereby affecting their function (159–162). Given their capacity to influence and communicate with neural cells, platelets and their secreted microparticles could also be engineered as drug carriers for the treatment of neurodegenerative disorders. However, until the exact mechanisms of the specific cell-cell communication between platelets and brain cells are fully understood, the value of this approach remains speculative. Furthermore, in order to develop human therapies with drug-loaded blood cells, extensive studies are needed to establish clinical grade protocols which standardize the varying methods of isolation and storage of platelets and platelet microparticles prior to their regulated reintroduction into individuals. Drug loading protocols for these natural vehicles, in terms of their capacity and compatibility with the drugs required to target neurodegenerative phenotypes, also need to be established. Nonetheless, in the field of regenerative medicine, considerable headway has already been made toward engineering extracellular vesicles and blood cell-inspired nanoparticles for therapeutic use (163–166).

## Conclusion

As summarized in this review, data connecting platelets and the factors they secrete to neurodegeneration have accumulated over recent years. However, it remains unclear whether platelet malfunction initiates the pathophysiological events that occur in neurodegenerative conditions, or whether platelet dysfunction arises as a consequence of other unfavorable changes that occur at early stages of these disorders. More data regarding the origin of platelet dysfunction are therefore required. During the onset of neurodegenerative conditions, factors released from healthy platelets could also have a protective role, as suggested by recent studies of AD (92) and cancer, where platelets initially suppress tumor angiogenesis (167). Moreover, platelets exhibit a sophisticated endocytic machinery (168) via which they could collect products that are released into the blood from other malfunctioning cells in an attempt to clear the systemic environment of cytotoxic components in the early stages of disease.

Although platelets and their released factors are gaining recognition for their potential therapeutic value in regenerative medicine, research is still in its infancy. Furthermore, the origin of platelets, the bone marrow, should not be overlooked, as a functional predisposition may also be inherited from their parent cells, the megakaryocytes. In conclusion, it remains highly interesting, but at the same time extremely challenging, to understand how platelets exert manifold actions across different tissues in physiological as well as pathological conditions. Their functional complexity clearly demands interdisciplinary approaches in order to develop novel therapeutic interventions which benefit from the multifaceted nature of platelets, including their capacity to facilitate crosstalk between the systemic environment and the brain.

## Author Contributions

OL and TW wrote the manuscript.

## Conflict of Interest

The authors declare that the research was conducted in the absence of any commercial or financial relationships that could be construed as a potential conflict of interest.

## Abbreviations

A_2A_R: adenosine A receptor
AD: Alzheimer's disease
ALS: amyotrophic lateral sclerosis
APP: amyloid precursor protein
EAE: experimental autoimmune encephalomyelitis
GABA: γ-aminobutyric acid
HD: Huntington's disease
mHtt: mutant huntingtin protein
MAO: monoamine oxidase
MPTP: 1-methyl-4-phenyl-1,2,3,6-tetrahydropyridine
MS: multiple sclerosis
PD: Parkinson's disease
PrP: prion protein
TDP-43: TAR DNA-binding protein of 43kDa.
